# Comparison of high-power single-ring isolation and box isolation in elderly patients with persistent atrial fibrillation

**DOI:** 10.1186/s12877-025-05981-y

**Published:** 2025-05-16

**Authors:** Yanbin Song, Xiaofang Liang, Sheliang Xue, Bo Xu, Jianqiang Xiao, Wenhua Li

**Affiliations:** 1 Wujin Hospital Affiliated With Jiangsu University, No. 2, Yongning North Road, Changzhou, Jiangsu 213017 China; 2 The Wujin Clinical College of Xuzhou Medical University, Changzhou, China

**Keywords:** Pulmonary vein isolation, Persistent atrial fibrillation, Single-ring isolation, Box isolation, Gastroesophageal reflux disease, Recurrence

## Abstract

**Background:**

This study evaluated the superiority of single-ring isolation (SRI) over box isolation using high power in elderly patients with persistent atrial fibrillation (PeAF).

**Methods:**

We retrospectively studied elderly patients with PeAF who underwent primary catheter ablation. The patients were divided into the SRI group and the posterior box isolation (BOXI) group. Basic characteristics, procedural variables, complications and atrial arrhythmia recurrence rates were collected.

**Results:**

Forty-five pairs of patients in the two groups were matched by 1:1 PSM. Compared with that in the BOXI group, the total procedure time in the SRI group was not significantly different (*P* = 0.340). However, there were significant reductions in the total ablation time, ring ablation lesions and number of lesions on the posterior wall in patients who underwent SRI compared to those who underwent BOXI (all *P* < 0.001). The cardiac troponin level in the SRI group was significantly lower (*P* = 0.023). There were significantly fewer mismatched three-dimensional mapping models and mismatched models per patient due to pain-induced movement in the SRI group (all *P* < 0.05). The questionnaires revealed that the pain score was significantly lower in the SRI group than in the BOXI group (*P* < 0.001). In addition, significantly fewer patients with SRI than with BOXI experienced gastrointestinal symptoms after the procedure (15.56% vs. 37.78%, *P* = 0.017). K‒M analysis revealed no significant difference in atrial arrhythmia-free survival at 12 months between the SRI and BOXI patients (*P* > 0.05).

**Conclusions:**

High-power SRI is safe and feasible and may be superior to the BOXI for experience of elderly patients with PeAF.

## Introduction

Pulmonary vein isolation (PVI) is the cornerstone for atrial fibrillation (AF) catheter ablation based on the crucial observation of electrical triggers of AF in the pulmonary veins (PVs) [1]. However, PVI alone seems insufficient, with lower rates of long-term success for nonparoxysmal AF, and further modification of the atrial substrate may be necessary [2]. Electrical isolation of the posterior wall (PW) is an important aspect of the management of persistent AF (PeAF) by electrophysiologists [3], which not only reduces the critical mass for the maintenance of AF but also decreases the number of rotors and multiple wavelets in the anterior wall, inferior wall and left atrium (LA) appendages during PeAF [4]. Posterior box isolation (BOXI) or single-ring isolation (SRI) can be applied to electrically isolate the PW. A recent study revealed that posterior BOXI for patients with PeAF resulted in significant left atrial reverse remodelling with a high rate of clinical success [5]. However, the available evidence suggests that BOX ablation can also cause damage to the oesophagus or vagal branches of the stomach [6, 7] and even catastrophic complications, such as atrio-oesophageal fistula [8–11].

SRIs are carried out based on physiological principles to electrically isolate the posterior LA and PVs. This approach limits the amount of ablation in the posterior LA, shortens the ablation time and has the potential to reduce collateral damage to the oesophagus, preventing the formation of atrial oesophageal fistula [12, 13]. Recently, high power (HP, 40–50 W) has been shown to create continuous and transmural lesions and has been demonstrated to be safe and effective in terms of clinical outcomes and lesion markers compared to conventional ablation with low power [14]. However, BOX ablation with HP still causes intractable intraoperative pain and oesophageal and vagal branch injury, especially in elderly patients, due to their greater susceptibility to gastroesophageal reflux disease.

To date, the efficacy of SRI ablation with a power of 40–45 W in elderly patients with PeAF remains unknown. Therefore, the aim of this study was to determine the feasibility and safety of SRI with inferior line sparing at high power for elderly patients with PeAF.

## Materials and methods

### Study population

A total of 501 consecutive patients with drug-refractory and symptomatic AF were retrospectively enrolled in this study. The patients were admitted to our centre and received first-time RFCA for AF from October 2017 to February 2023 at Wujin People’s Hospital. The exclusion criteria included (1) age < 65 years; (2) paroxysmal atrial fibrillation; (3) a history of RFCA for AF; (4) other RFCA strategies; (5) duration of atrial fibrillation > 10 years; (6) left atrial diameter (LAD) > 56 mm; and (7) lack of essential data.

The enrolled patients were divided into the BOXI group and the SRI group according to the ablation approach used to treat AF. In the SRI group, patient enrollment primarily occurred between December 2021 and January 2023. During this period, the SRI procedure for all eligible patients with PeAF were consistently performed by two experienced electrophysiologists of our centre.

Our study protocol was performed in accordance with the Helsinki Declaration and was approved by the Ethics Committee of Wujin Hospital Affiliated with Jiangsu University (2023-SR-055).

### Variables and definitions

Basic characteristics, including sex, age, body mass index (BMI), history of hypertension, type 2 diabetes mellitus (T2DM), coronary artery disease (CAD), ischaemic stroke, left atrium diameter (LAD), left ventricular ejection fraction (LVEF), and CHA2DS2-VASc score, were collected from the electronic medical records of all included patients. Variables of the ablation procedure, including the total procedure time, total ablation time, total ablation lesions, initial ring lesions, visual analogue scale (VAS) score and complications, were collected. Venous blood samples were obtained from all patients in a fasting state on the morning following admission. Plasma lipid parameters, including total cholesterol (TC), triglyceride (TG), high-density lipoprotein cholesterol (HDL-C) and low-density lipoprotein cholesterol (LDL-C), were evaluated using standard techniques. Venous blood for detecting cardiac troponin (cTnI) was collected 24 h after the procedure.

The VAS score [15] was used to rate pain during entire ablation process with a 10-cm straight line divided into 10 equal marks, with 0 indicating no pain and 10 indicating intolerable pain.

Mismatch of three-dimensional (3D) mapping models was defined as displacement and transient procedure interruption resulting from pain-induced body movement.

### Catheter ablation procedure

Procedures were performed under conscious sedation using intravenous fentanyl for anaesthesia. A decapolar catheter (APT Medical, China) was placed in the coronary sinus. After double transseptal punctures, a deflectable PentaRay mapping catheter (D128211, Biosense Webster, US) and a 3.5 mm 56-well open irrigated-tip ablation catheter (Thermocool SF, Biosense Webster, US) were advanced into the LA for mapping and ablation via the femoral veins.

3D electroanatomical geometries of the LA and PVs were reconstructed using the pentaray catheter in conjunction with the Carto 3 mapping system after selective PV angiography. Point-by-point PVI was performed using RF energy and a 3.5 mm 56-well irrigated-tip ablation catheter. Intravenous heparin was administered to maintain an activated clotting time of 300 to 350 s. Ablation was performed with an interlesion distance ≤ 5 mm, a power of 40–45 W, a temperature of 43 °C, a flow rate of 15 to 18 mL/min and an ablation index (AI) of 350–380 at the posterior wall and 450–500 at the anterior wall and roof wall. In addition, continuous infusions of heparinized saline were connected to the transseptal sheaths to avoid thrombus or air embolism during the procedures.

**Fig. 1:**
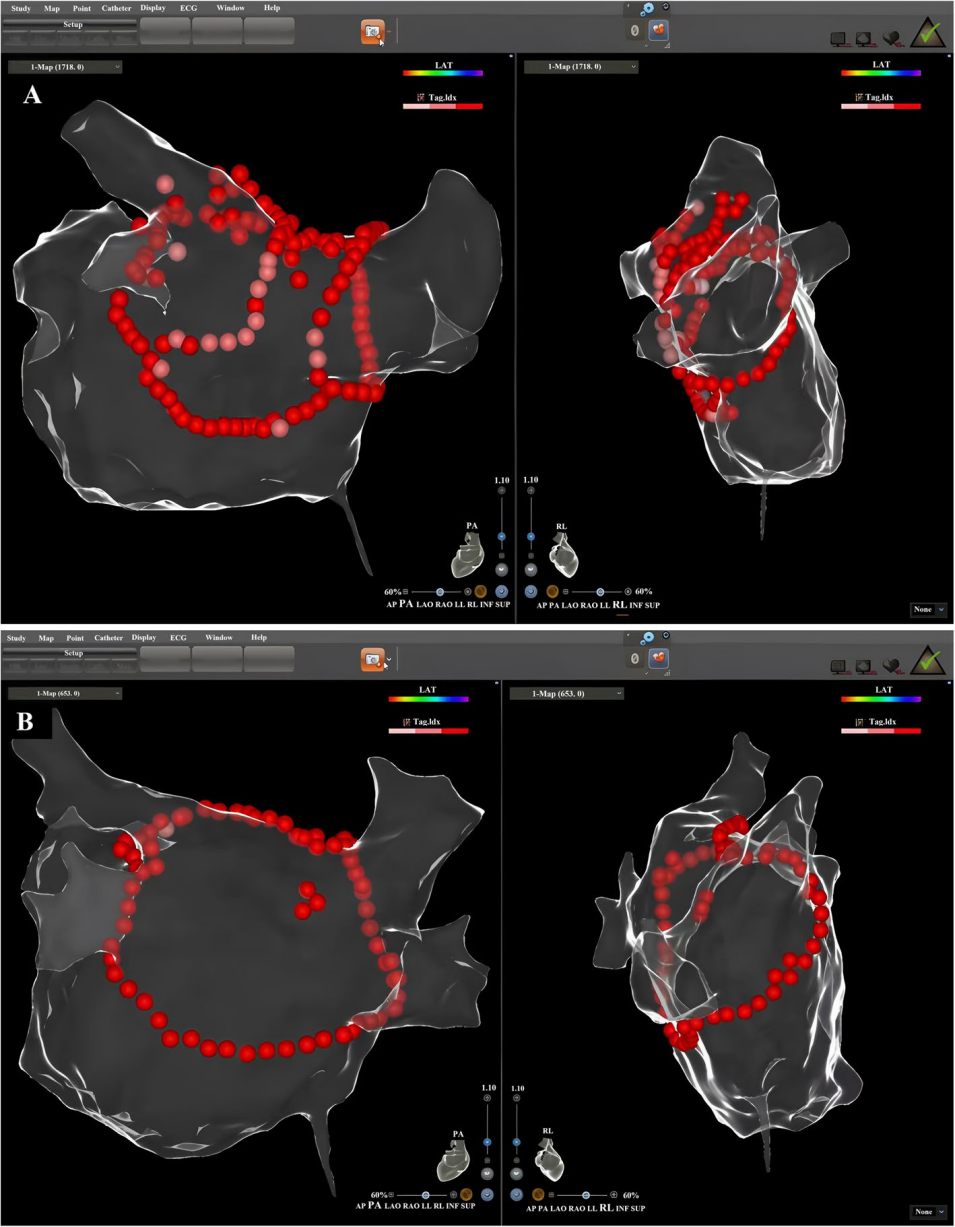
**A** In addition to PVIs, BOXI involves two linear ablation lines (roof and inferior) connecting the superior and inferior PVs, forming a box-shaped isolation zone (red dots). **B** SRI creates a single continuous circular lesion encircling all four PVs and the posterior LA (red dots), requiring fewer ablation lesions in the posterior LA due to its anatomical efficiency

PVI was performed with ipsilateral PV isolation in pairs, with an entrance and exit block as the electrophysiological endpoint [16]. In BOXI ablation, PVIs were performed first, followed by linear ablation at the roof and inferior lines connecting the superior and inferior PVs [5]. These lines, combined with bilateral PV isolation, form a “box” around the posterior left atrium (LA) (Fig. 1A). The SRI was performed as follows: anterior to right pulmonary veins, roof, left pulmonary vein ridge, and finally connecting both the inferior PVs with a V-shaped line. This approach creates a continuous circular ablation line encircling all four PVs and the posterior LA in a single loop (Fig. 1B).

When bidirectional conduction block was not achieved, point-by-point activation was performed to identify the earliest breakthrough with the mapping catheter within the ring until all the gaps were abolished.

After ablation, if sinus rhythm was not achieved, a temporary intravenous dose of 1–2 mg of midazolam was administered, followed by external application of ≤ 3 synchronized, biphasic direct current shocks (200 J) to restore sinus rhythm.

The electrical isolation criteria for PW and PVs included [17] (1) dissociation or absence of electrical activity within the ring, (2) the remaining atrium was not captured during pacing of the PW and PVs in sinus rhythm, and (3) associated electrical activity in the PW or PVs was absent when pacing from the coronary sinus catheter.

### Postablation Management and follow-up

Patients were scored for intraoperative pain using the VAS on the day after the ablation procedure. After the ablation procedure, intravenous heparin was administered for 1 day to all patients, followed by treatment with warfarin or new oral anticoagulation agents for at least 3 months according to the CHA2DS2-VASc score. All patients were kept on antiarrhythmic drugs for 1 month after ablation unless contraindications were present. The patients were routinely prescribed proton pump inhibitors for 4 weeks after ablation. The patients were followed up at 1, 3, 6 and 12 months after ablation by the referring cardiologists. All patients underwent 24-h Holter recording to determine the incidence of postprocedural atrial arrhythmia at 3, 6 and 12 months after ablation. Recurrence was defined as ≥ 30 s of any atrial arrhythmia, including AF and atrial tachycardia (AT), after a 90-day blanking period.

### Statistical analysis

Statistical analysis was performed using SPSS 25.0. Continuous variables were expressed as the mean ± SD, and nonnormally distributed variables were expressed as the median (interquartile range). Categorical variables were presented as frequencies and percentages. Continuous variables were compared by Student’s t test, one-way ANOVA or the nonparametric Mann‒Whitney test, as appropriate. Differences between the two groups were tested using the chi-square test for categorical variables. Recurrence rates during the 12-month follow-up were compared using Kaplan‒Meier analysis. PSM was performed to minimize potential confounding variables. One-to-one nearest-neighbour matching was conducted using a match tolerance of 0.1. All *P* values were two-sided. A *P* value less than 0.05 was considered to indicate statistical significance.

## Results

**Fig. 2:**
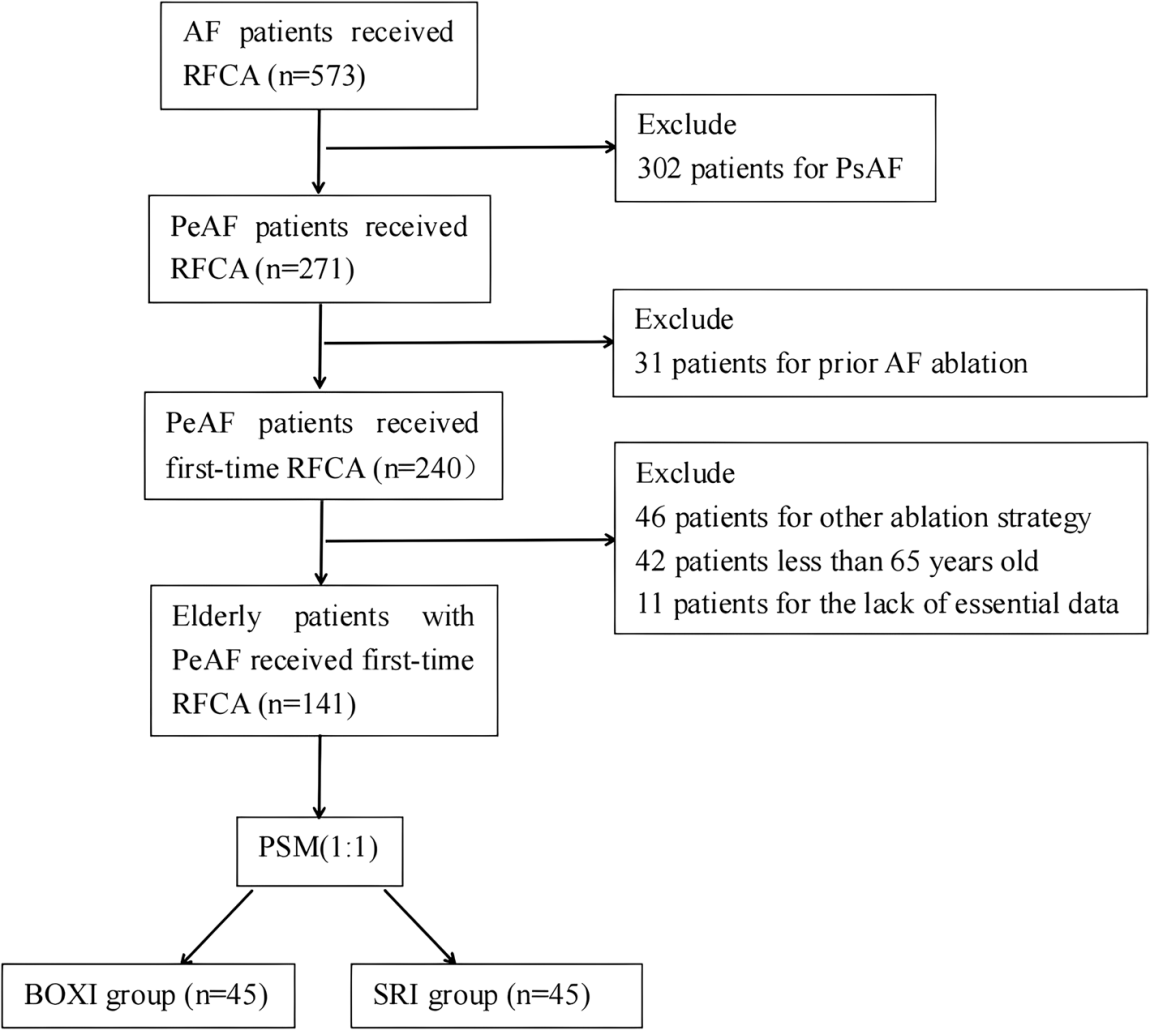
Study flowchart. AF, atrial fibrillation; RFCA, radiofrequency catheter ablation; PsAF, paroxysmal atrial fibrillation; PeAF, persistent atrial fibrillation; PSM, propensity score matching; BOXI, box isolation; SRI, single-ring isolation

A total of 501 patients underwent RFCA for AF from October 2017 to February 2023 at our hospital. Based on the inclusion and exclusion criteria, a total of 141 patients (*n* = 83 for the BOXI group; *n* = 58 for the SRI group) were enrolled for further analysis (Fig. 2).

### Baseline characteristics of the two groups

**Fig. 3:**
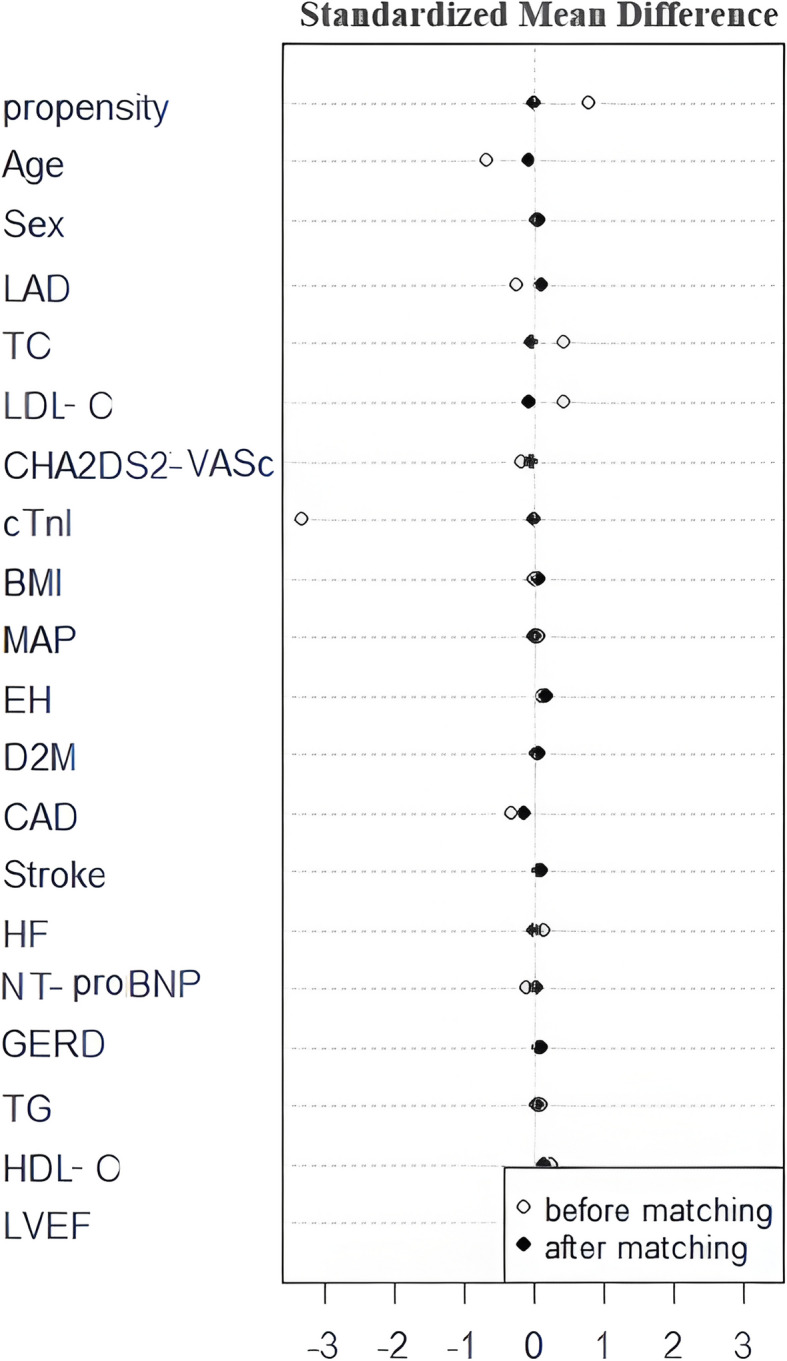
The clinical baseline data of 19 confounding variables before and after matching between the two groups. LAD, left atrium diameter; TC, total cholesterol; LDL-C, low-density lipoprotein cholesterol; cTnI, cardiac troponin; BMI, body mass index; MAP, mean arterial pressure; EH, essential hypertension; D2M, type 2 diabetes mellitus; CAD, coronary artery disease; HF, heart failure; NT-proBNP, N-terminal pro-B type natriuretic peptide; GERD, gastroesophageal reflux disease; TG, triglyceride; HDL-C, high-density lipoprotein cholesterol; LVEF, left ventricular ejection fraction

The present study included patients [(69 (48.94%) males; 73.15 ± 4.96 years)] with highly symptomatic PeAF before PSM. Some of the differences in the baseline characteristics between the two groups before and after matching are summarized in Table 1. There were significant differences in age and TC and LDL-C levels between the two groups before matching. In our study, a standardized mean difference less than 10% demonstrated a sufficient balance of covariate distribution and high-quality matching between the two groups (Fig. 3). A total of 45 pairs of patients were successfully matched in the two groups by 1:1 PSM. No significant differences were observed in the confounding variables between the two groups after PSM (Fig. 3, Table 1).

**Table 1:** Comparison of patient characteristics between the two groups before and after propensity score matching

Characteristics	Before matching			After matching		
	**SRI group (*n* = 58)**	**BOXI group (*n* = 83)**	***P* value**	**SRI group (*n* = 45)**	**BOXI group (*n* = 45)**	***P* value**
Age, years	71.28 ± 4.73	74.46 ± 4.71	< 0.001	72.58 ± 4.52	72.93 ± 3.75	0.685
male, n (%)	30 (51.72)	39(46.99)	0.580	24(53.33)	23 (51.11)	0.833
BMI, kg/m^2^	24.89 ± 3.70	24.89 ± 3.63	0.990	24.95 ± 3.72	24.70 ± 3.14	0.728
MAP, mmHg	97.16 ± 13.14	96.13 ± 13.21	0.650	95.38 ± 13.26	95.51 ± 11.32	0.961
Hypertension, n (%)	40 (68.97)	61 (73.49)	0.557	32 (71.11)	36 (80.00)	0.327
T2DM, n (%)	16 (27.59)	20 (24.09)	0.640	13 (28.89)	12 (26.67)	0.814
CAD, n (%)	8 (13.79)	20 (24.09)	0.131	7 (15.56)	9 (20.00)	0.581
Stroke, n (%)	8 (13.79)	8 (9.64)	0.444	6 (13.33)	5 (11.11)	0.748
HF, n (%)	34 (58.62)	55(66.27)	0.355	25(55.56)	25 (55.56)	1.000
LAD, mm	44.24 ± 5.08	45.54 ± 4.67	0.119	44.84 ± 4.52	44.27 ± 4.36	0.539
LVEF, %	56.64 ± 9.33	55.67 ± 7.81	0.507	56.02 ± 9.65	56.42 ± 6.90	0.822
CHA2DS2-VASc score	3.72 ± 1.44	3.96 ± 1.40	0.324	3.71 ± 1.41	3.76 ± 1.38	0.880
GERD, n (%)	12 (20.69)	14 (16.87)	0.565	7 (15.56)	8 (17.78)	0.777
TC, mmol/L	4.23 ± 0.93	3.84 ± 1.02	0.022	4.04 ± 1.07	4.01 ± 0.83	0.852
TG, mmol/L	1.31(0.88, 1.75)	1.29 (0.98, 1.86)	0.803	1.22 (0.95, 1.78)	1.31 (0.99, 1.76)	0.678
HDL-C, mmol/L	1.38 ± 0.43	1.27 ± 0.36	0.103	1.37 ± 0.46	1.30 ± 0.37	0.479
LDL-C, mmol/L	2.47 ± 0.72	2.17 ± 0.75	0.019	2.28 ± 0.67	2.32 ± 0.80	0.819
cTnI, ng/ml	0.012 (0.012, 0.012)	0.012 (0.012, 0.012)	0.969	0.012 (0.012, 0.012)	0.012 (0.012, 0.012)	0.207
NT-proBNP, pg/ml	1030.00 (422.00, 1860.00)	562.50 (427.25, 1280.00)	0.111	558.00 (422.50, 1130.00)	977.99 (372.50, 1850.00)	0.272

Values are presented as the mean ± SD, median (interquartile range), or number (%)

A* P* value < 0.05 was considered to indicate statistical significance

*BMI* Body mass index, *MAP* Mean arterial pressure, *T2DM* Type 2 diabetes mellitus, *CAD* Coronary artery disease, *HF* Heart failure, *LAD* Left atrium diameter, *LVEF* Left ventricular ejection fraction, *CHA2DS2-VASc score* Stroke risk score, *GERD* Gastroesophageal reflux disease, *TC* Total cholesterol, *TG* triglyceride, *HDL-C* High-density lipoprotein cholesterol, *LDL-C* Low-density lipoprotein cholesterol, *cTnI* Cardiac troponin I, *NT-proBNP* N-terminal pro-B type natriuretic peptide

### Differences in procedure-related variables between the two groups after PSM

Electroanatomic maps of the LA in posteroanterior and right lateral views for the two ablation methods are shown in Fig. 1a and b, respectively. Posterior left atrium isolation was achieved, and AF was terminated after the procedure in all patients. The differences in procedure-related variables between the two groups were examined and are summarized in Table 2. The total procedure time did not differ between the SRI group and the BOXI group (*P* = 0.340). However, the procedure time from the 26th case to the 45th case in the SRI group was significantly shorter than that in the BOXI group (186.04 ± 38.69 vs. 224.60 ± 49.79, *P* = 0.004). The total ablation time in patients who underwent SRI was significantly shorter than that in patients who underwent BOXI (55.23 ± 9.93 min vs. 68.97 ± 13.59 min, *P* < 0.001). Notably, the average ablation time from the 26th case to the 45th case with SRI was only 47.92 ± 4.43 min. There were significantly fewer ring ablation lesions in the SRI group than in the BOXI group (*P* < 0.001). Moreover, the number of lesions on the posterior wall in the SRI group was significantly lower than that in the BOXI group (*P* < 0.001). There was no significant difference in the first pass isolation rate between the SRI group and the BOX group (71.11% vs. 75.55%, *P* = 0.634). In addition, the cTnI level in the SRI group was significantly lower than that in the SRI group (*P* = 0.023). The 3D mapping models were mismatched due to pain-induced movement in significantly fewer patients in the SRI group (8.89% vs. 26.67%, *P* = 0.027) (Table 3). The average number of mismatched 3D mapping models per patient was also significantly lower in the SRI group (*P* = 0.025).

**Table 2:** Procedural characteristics of the matched groups

Variables	SRI group (*n* = 45)	BOXI group (*n* = 45)	*P* value
Procedure time, min	210.00 ± 47.70	220.16 ± 52.54	0.340
Procedure time (from the 26th case to the 45th case), min	186.04 ± 38.69	224.60 ± 49.79	0.004
Total ablation time, min	55.23 ± 9.93	68.97 ± 13.59	< 0.001
Ablation time (from the 26th case to the 45th case), min	47.92 ± 4.43	66.08 ± 10.46	< 0.001
Total ablation lesions	104.20 ± 14.79	126.73 ± 18.66	< 0.001
Ablation lesions of PW	17.78 ± 4.88	40.16 ± 10.24	< 0.001
First pass isolation rate, n (%)	32 (71.11)	34 (75.56)	0.634
Immediate sinus rhythm	14 (31.11)	6 (13.33)	0.043
DC shocks, n (%)	31 (68.89)	39 (86.67)	0.043
Number of DC shocks	0.93 ± 0.54	0.69 ± 0.51	0.030
cTnI, (ng/ml)	2.54 ± 0.99	2.06 ± 0.98	0.023

Values are presented as the mean ± SD, median (interquartile range), or number (%)

A *P* value < 0.05 was considered to indicate statistical significance

*PW* Posterior wall, *DC* Direct current, *cTnI* Cardiac troponin I

**Table 3:** Comparison of procedure-related pain scores and complications between the matched groups

Variables	SRI group (*n* = 45)	BOXI group (*n* = 45)	*P* value
VAS score	3.40 ± 1.05	5.53 ± 1.27	< 0.001
Mismatch of 3D mapping models due to pain-induced movement, n (%)	4 (8.89%)	12 (26.67%)	0.027
Number of mismatched 3D mapping models per patient due to pain-induced movement	0 (0, 0)	0 (0, 1)	0.025
Gastrointestinal symptoms, n (%)	7 (15.56)	17(37.78)	0.017
Fever, n (%)	2 (4.44)	5 (11.11)	0.434
Death, n (%)	0	0	-
atrioesophageal fistula, n (%)	0	0	-
cardiac perforation, n (%)	0	0	-

Values are presented as the mean ± SD, median (interquartile range), or number (%)

A *P* value < 0.05 was considered to indicate statistical significance

*VAS* Visual analogue scale, *3D* three-dimensional

In addition, immediate sinus rhythm was achieved in 15 (33.33%) patients who underwent SRI after ablation but in only 7 (15.56%) patients who underwent BOXI (*P* = 0.043). Similarly, < 2 synchronized, biphasic direct current (DC) shocks were used to restore sinus rhythm in 64 patients: 29 (64.44%) in the SRI group and 35 (77.78%) in the BOXI group. The proportion of patients who needed DC shocks to terminate AF were significantly lower in patients with SRI than in those with BOXI (*P* < 0.05).

### Comparison of procedure-related pain scores and complications between the matched groups

Pain scores during the procedure were retrospectively investigated from patients using VAS by the cardiologists on the day after ablation. The results of the questionnaires revealed that pain scores were significantly lower in the SRI group than in the BOXI group (3.40 ± 1.05 vs. 5.53 ± 1.27, *P* < 0.001).

No major procedure-related complications, including death, cardiac perforation, or atrioesophageal fistula, occurred in any of the study patients. Fever occurred in 7 patients overall; 2 (4.44%) and 5 (11.11%) in the SRI and BOXI groups, respectively. In addition, 7 patients in the SRI group (15.56%) and 17 patients in the BOXI group (37.78%) experienced gastrointestinal symptoms after the procedure, including chest pain, nausea, and vomiting (*P* = 0.017) (Table 3).

### Atrial arrhythmia–free survival rates

**Fig. 4:**
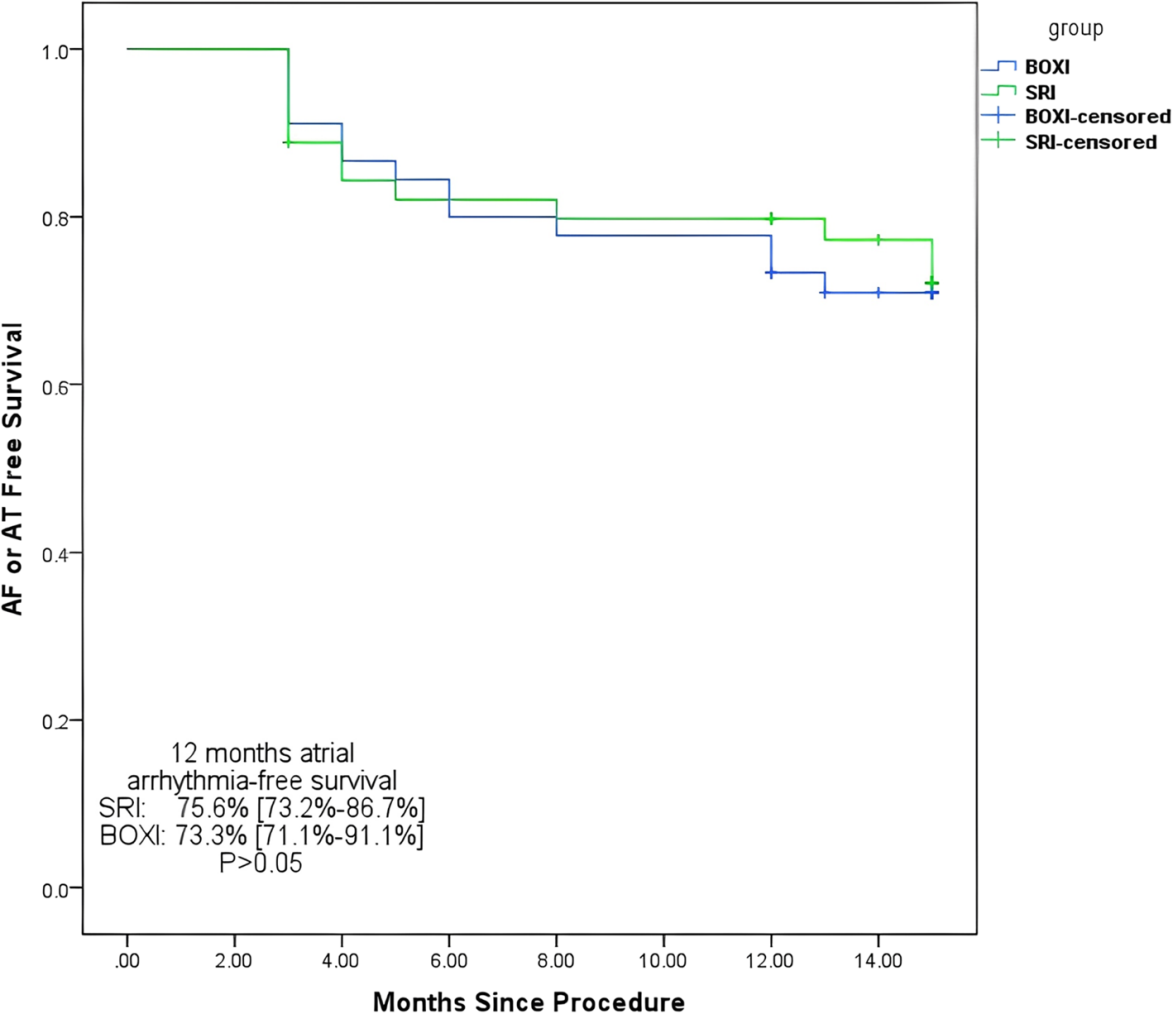
Kaplan–Meier plots of atrial arrhythmia-free survival. K‒M survival plots comparing atrial arrhythmia (atrial fibrillation or atrial tachycardia)-free survival between the single-ring isolation (SRI) group and the box isolation (BOXI) group on the posterior wall of the left atrium

The mean follow-up period was not significantly different between the SRI group and the BOXI group [13.42 ± 1.69 months vs. 13.09 ± 1.49 months, *P* > 0.05]. No patients underwent a second ablation procedure during the 90-day recovery period. At the most recent follow-up visit, AF recurrence was detected in 6 patients in the SRI group and 7 patients in the BOXI group. Eight additional patients with AT in each group were observed. The pattern of atrial arrhythmia–free survival is shown in Fig. 4 by Kaplan‒Meier curves. No significant difference was noted in atrial arrhythmia-free survival at 12 months for SRI and BOXI patients (12-month atrial arrhythmia-free survival: 75.6% [73.2%-86.7] for SRI vs. 73.3% [71.1%-91.1%] for BOXI; *P* > 0.05) (Fig. 4).

## Discussion

**Fig. 5:**
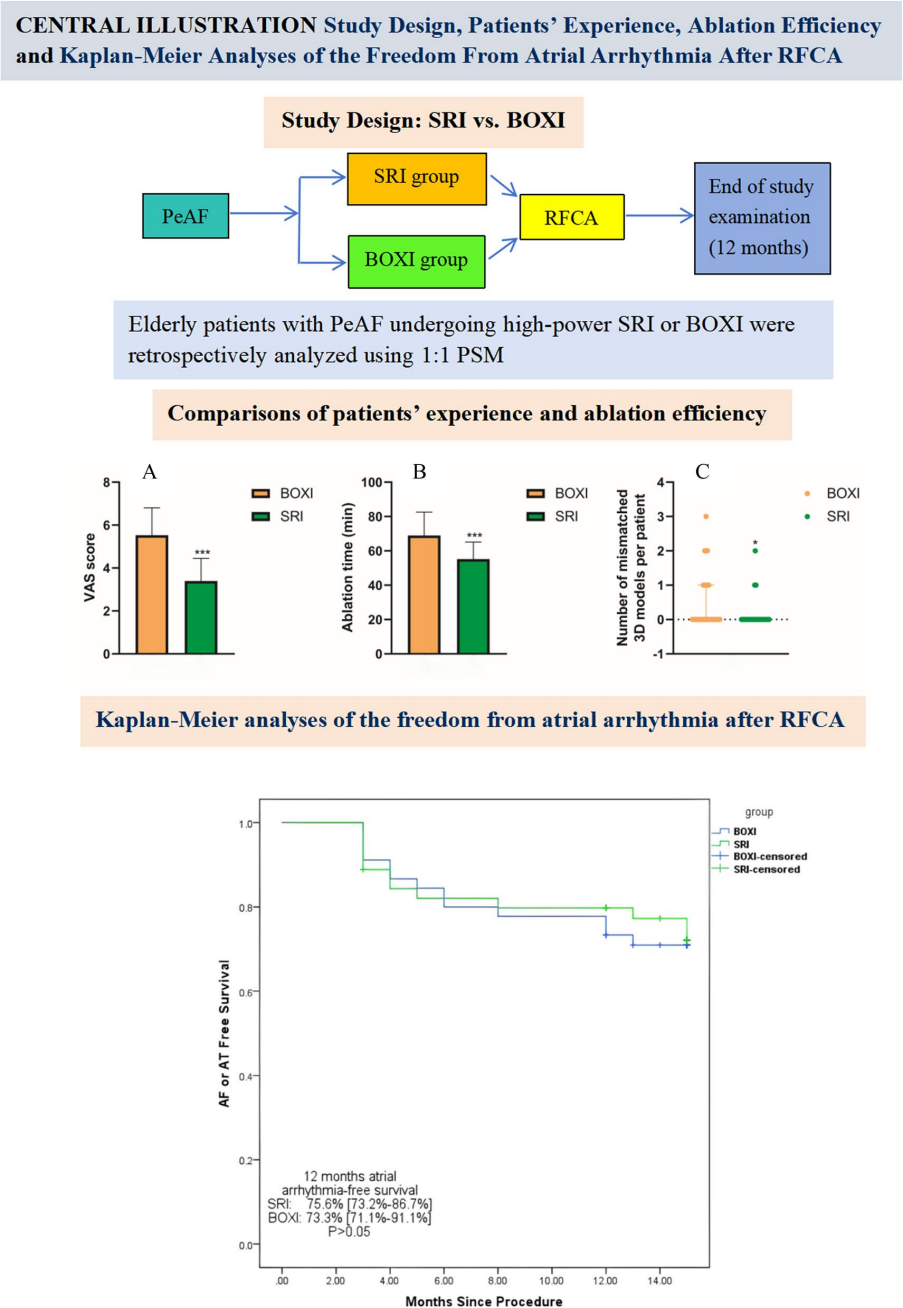
Central Illustration. Compared with BOXI, there were significant reductions in the total ablation time (**A**), VAS score (**B**) and number of mismatched 3D mapping models per patient due to pain-induced movement (**C**) in SRI. ***, *P* < 0.001; *, *P* < 0.05; BOXI = posterior box isolation; SRI = single-ring isolation; PeAF = persistent atrial fibrillation; RFCA = radiofrequency catheter ablation; PSM = propensity score matching

In this single-centre, retrospective study, we investigated differences between SRI and BOXI with high power in elderly patients with PeAF in terms of efficacy and safety. There were four key findings. First, the total ablation time, number of ablation lesions and extent of myocardial injury in the SRI group were significantly lower than those in the BOXI group. Second, the proportion of patients with immediate sinus rhythm was significantly greater in the SRI group during the ablation procedure. Third, a significant reduction in procedure-related gastrointestinal symptoms, pain scores and mismatched 3D mapping models due to pain-induced movement was observed in patients who underwent SRI compared to those who underwent BOXI. Fourth, the 12-month atrial arrhythmia recurrence rate did not differ significantly between the two groups. The study design and main findings were showed in the Central illustration (Fig. 5).

The prevalence of gastroesophageal disorders, such as gastroesophageal reflux disease (GERD), has been reported to markedly increase with age [18]. A previous study showed that the incidence of GERD was as high as 23% in elderly residents of American nursing facilities [19]. GERD was reported to be associated with oesophageal injury after RFCA [12]. Therefore, reduced ablation of the PW in elderly patients may be an important strategy to avoid oesophageal injury. Similarly, in our study of elderly patients with AF, there was also a high rate of GERD (16.67%). Therefore, greater concern regarding oesophageal injury in PW ablation should be directed towards this patient population. Previous studies have demonstrated that the SRI plays an important role in decreasing the ablation time [12, 13]. The findings of the present study align with this, as the ablation time of the SRI group was significantly shorter than that of the BOXI group (*P* < 0.001). Compared with the BOXI technique, the SRI technique eliminates the need to create two additional ablation lines between the roof and inferior aspects of the posterior wall. This results in a significant reduction in total posterior wall ablation lesions (SRI: 17.78 ± 4.88 vs. BOXI: 40.16 ± 10.24; *P* < 0.001). The reduced lesion count in SRI aligns with its physiological design to isolate the PVs and posterior LA with a single, contiguous line rather than multiple interconnected lines, potentially lowering the risk of esophageal injury associated with energy delivery near the esophagus.

Nevertheless, regarding total procedure time, our results failed to show a significant difference between the groups. These results are similar to those of a previous report in which PVI was achieved with a similar procedure time [20]. It is difficult for inexperienced operators to achieve the isolation of PVs and PW by a single ring without additional segmental lesions within the ring, which may increase the procedural time. Some anatomical considerations for successful SRI should be considered. The left atrial diverticulum, a characteristic anatomical structure of the LA, is often adjacent to the right superior pulmonary vein. However, due to the insertions of fibres from the epicardium, achieving SRI may pose challenges if the roof line is anterior to the diverticulum. In addition, an anterior roof line may transversely cross the Bachmann bundle, causing difficulties in achieving SRI and requiring additional ablation within the ring. Ablation along the inferior line is recommended as the final step. With robust anterior wall and top line ablation, the PW may only require minimal ablation, such as inferior line sparing, to achieve overall isolation of the pulmonary veins and PW, which can significantly reduce oesophageal injury [21]. In our study, the opportunity to practice this technique more (> 20 patients) may have improved the efficiency of SRI and reduced the procedural time, which is evident from the fact that the procedure time for the last 25 patients in the SRI group was significantly shorter than that in the BOXI group (186.04 ± 38.69 min vs. 224.60 ± 49.79 min, *P* = 0.004). Hence, the SRI may be superior in terms of total procedure time for the majority of elderly patients, especially for elderly patients with poor surgical tolerance.

Patients who undergo AF ablation often experience pain, especially during PW ablation. Even with the use of morphine and fentanyl for analgesia, some elderly patients still experience intolerable pain, which not only greatly reduces the comfort and satisfaction of patients with the operation but also interrupts the procedure due to the mismatch of the 3D model caused by movement, resulting in insufficient local ablation and increased procedural time, thereby increasing the risk of recurrence. General anaesthesia-based AF catheter ablation is an ideal method, but it is difficult to implement in many centres due to the lack of anaesthesiologists. Therefore, the SRI with minimal PW ablation is the best option. We evaluated the patients' experience during the procedure using retrospective VAS questionnaires. The results showed that elderly patients in the SRI group had lower VAS than those in the BOXI group (3.40 ± 1.05 vs. 5.53 ± 1.27, *P* < 0.001), suggesting greater procedural comfort and satisfaction, which may enhance the overall ablation experience.

During AF catheter ablation, cTnI may increase, reflecting the extent and scope of myocardial injury caused by ablation. Our study showed that troponin I levels were significantly lower in the SRI group than those in the BOXI group at 24 h postablation (2.54 ± 0.99 ng/ml vs. 2.06 ± 0.98 ng/ml, *P* = 0.023), which indicates that SRI is associated with less myocardial injury of PW and potential oesophageal injury than the BOXI.

The high-power short-duration (HPSD) ablation strategy comprises the use of higher RFCA power (≥ 40 W) and a shorter duration (5–15 s) of each RF energy application, and HPSD ablation results in larger lesion diameters and smaller lesion depths than conventional (20–35 W, 10–30 s) applications [22]. High-power ablation was previously associated with better procedural effectiveness than conventional RFCA with low power [23]. Currently, there are relatively few reports on the use of HPSD ablation for SRI in elderly patients. Our study revealed that SRI combined with HPSD demonstrated greater effectiveness in restoring sinus rhythm than did BOXI alone. Moreover, the proportion of DC shocks to terminate atrial arrhythmia in patients who underwent SRI was significantly lower than that in patients who underwent BOXI (*P* = 0.043). Obviously, the SRI procedure with HPSD was more effective and comfortable for elderly patients. The mechanism for the higher percentage of immediate sinus rhythm in patients with SRI is unclear. A possible explanation is that with the intervention of left atrial substrates in the same range, less PW injury and pain stimulation during HPSD ablation may reduce the secretion of adrenergic hormones and stimulation of peripheral ganglia of the heart, lower neural excitability, prolong the effective refractory period of atrial muscle, and improve electrical remodelling of atrial muscle so that AF cannot be maintained.

Prior studies have suggested that fever (73%), neurological (72%), gastrointestinal (41%), and cardiac (40%) symptoms are the most common adverse events that occur between 0 and 60 days postablation (median 21 days). Moreover, atrioesophageal fistula complicating AF ablation is associated with high mortality [24]. In our study, radiofrequency energy was applied at each point with a power of 40–45 W around the PV and near the oesophagus. No major procedure-related complications, including death, cardiac perforation, or atrioesophageal fistula, occurred in any of the study patients. In addition, compared with those in the BOXI group, procedure-related gastrointestinal symptoms were reduced in the SRI group after ablation (15.56% vs. 37.78%, *P* = 0.017), which may be attributed to fewer PW interventions and oesophageal injury, highlighting the superiority of SRI. Overall, the above results suggest that it is safe to isolate the pulmonary veins and posterior left atrium with the SRI technique using high power in elderly patients.

Long-term results after pulmonary vein isolation have demonstrated high rates of recurrent arrhythmia after ablation procedures. The arrhythmia-free survival rates after catheter ablation procedures were 40%, 37%, and 29% after 1, 2, and 5 years, respectively [25]. A meta-analysis revealed that wider isolation techniques had lower recurrence rates than did ostial isolation in both paroxysmal and persistent AF patients [26]. In addition, Lim et al. [27] reported the superiority of SRI over wide antral pulmonary vein isolation regarding AF recurrence in patients with symptomatic AF (61% paroxysmal, 39% persistent/longstanding persistent). They showed that AF-free survival at 2 years was better after single-ring isolation (74% [95% CI, 65%–82%]) than wide antral isolation (61% [51%–70%]; *P* = 0.031). Our research was based on the comparison of the long-term outcomes of two ablation approaches with the same electrical isolation range. K‒M analysis revealed that the SRI did not improve AF-free survival or AT-free survival at 12 months (atrial arrhythmia-free survival: 75.6% [73.2%-86.7%] for SRI vs. 73.3% [71.1%-91.1%] for BOXI, *P* > 0.05). One explanation for this difference is that the proportion of PeAF significantly differed between our study and the study by Lim et al. (100% vs. 39%, *P* < 0.01). Wide antral pulmonary vein isolation may not be sufficient for PeAF, and other ablation strategies are needed, such as posterior box isolation and substrate and trigger ablation [28]. The posterior wall of the LA is isolated in the SRI, which reduces the critical substrate for the maintenance of PeAF and may improve the outcome [4]. However, in our study, the scope of LA isolation was the same between SRI and BOXI, indicating that the areas of ablation targeted to eliminate triggers and substrates for AF maintenance were also the same. This may be the reason for the lack of a significant difference in long-term efficacy between our two isolation methods. Furthermore, the patients in our study were followed for a relatively short period.

There are several limitations to this study that need to be considered. First, owing to the small sample size and retrospective nature of the study, our results could be biased even if PSM was performed to adjust for between-group differences in the baseline data. Second, gastrointestinal symptoms were not sufficient to evaluate the difference in oesophageal injury between the SRI and BOXI groups. Oesophageal temperature monitoring may demonstrate the advantages of SRI over BOXI. Third, the 12-month follow-up period was relatively short, and there were no long-term outcomes. Fourth, in our study, the VAS score assessed pain throughout the entire ablation process, not just during posterior wall ablation. It is well known that the patients' pain during the ablation mainly occurred during the intervention for posterior wall, especially in the lower posterior wall region of the left and right pulmonary veins. Certainly, we cannot overlook that a small subset of patients may experience maximum pain at times other than during posterior wall ablation. Capturing the patient's pain score during the posterior wall ablation procedure would yield more accurate data and enhance its persuasive value. Finally, because AF recurrence was assessed on the basis of symptoms and 24-h Holter, the occurrence of atrial arrhythmia may have been missed. Daily self-pulse checks, 7-day Holter monitoring and wearable monitoring devices should be used to evaluate AF recurrence in future studies.

In conclusion, this study demonstrates, for the first time, that an SRI ablation strategy with high power could be more efficient and safer for most elderly patients with PeAF than a BOXI ablation strategy. Our data provide a practical method of RFCA in elderly patients with PeAF, but future long-term investigations with larger sample sizes are necessary to confirm our findings.

## Data Availability

All the data used to support the findings of this study are available from the corresponding author upon reasonable request.

## References

[1] Haïssaguerre M, Jaïs P, Shah DC, et al. Spontaneous initiation of atrial fibrillation by ectopic beats originating in the pulmonary veins. N Engl J Med. 1998;339(10):659–66. 9725923 10.1056/NEJM199809033391003

[2] Tilz RR, Chun KR, Schmidt B, et al. Catheter ablation of long-standing persistent atrial fibrillation: a lesson from circumferential pulmonary vein isolation. J Cardiovasc Electrophysiol. 2010;21(10):1085–93. 20487116 10.1111/j.1540-8167.2010.01799.x

[3] Lall SC, Melby SJ, Voeller RK, et al. The effect of ablation technology on surgical outcomes after the Cox-maze procedure: a propensity analysis. J Thorac Cardiovasc Surg. 2007;133(2):389–96. 17258570 10.1016/j.jtcvs.2006.10.009

[4] Kumagai K, Toyama H, Ashihara T. Impact of box isolation on rotors and multiple wavelets in persistent atrial fibrillation. Circ J. 2020;84(3):419–26. 32051349 10.1253/circj.CJ-19-0826

[5] Miwa Y, Mohri T, Katsume Y, et al. Left atrial reverse remodeling following the modified box isolation with centerline in patients with persistent atrial fibrillation. Int Heart J. 2021;62(5):1005–11. 34544979 10.1536/ihj.21-108

[6] Cummings JE, Schweikert RA, Saliba WI, et al. Brief communication: atrial-esophageal fistulas after radiofrequency ablation. Ann Intern Med. 2006;144(8):572–4. 16618954 10.7326/0003-4819-144-8-200604180-00007

[7] Shah D, Dumonceau JM, Burri H, et al. Acute pyloric spasm and gastric hypomotility: an extracardiac adverse effect of percutaneous radiofrequency ablation for atrial fibrillation. J Am Coll Cardiol. 2005;46(2):327–30. 16022963 10.1016/j.jacc.2005.04.030

[8] MacDonald MR, Connelly DT, Hawkins NM, et al. Radiofrequency ablation for persistent atrial fibrillation in patients with advanced heart failure and severe left ventricular systolic dysfunction: a randomised controlled trial. Heart. 2011;97(9):740–7. 21051458 10.1136/hrt.2010.207340

[9] Michowitz Y, Rahkovich M, Oral H, et al. Effects of sex on the incidence of cardiac tamponade after catheter ablation of atrial fibrillation: results from a worldwide survey in 34 943 atrial fibrillation ablation procedures. Circ Arrhythm Electrophysiol. 2014;7(2):274–80. 24519888 10.1161/CIRCEP.113.000760

[10] Baman TS, Jongnarangsin K, Chugh A, et al. Prevalence and predictors of complications of radiofrequency catheter ablation for atrial fibrillation. J Cardiovasc Electrophysiol. 2011;22(6):626–31. 21235674 10.1111/j.1540-8167.2010.01995.x PMC3471534

[11] Mujovic NM, Marinkovic MM, Potpara TS, Geller L. Catheter ablation of lone atrial fibrillation. Curr Pharm Des. 2015;21(5):591–612. 25175086 10.2174/1381612820666140825144226

[12] Mahajan R, Thiyagarajah A, Lau DH, Sanders P. Single ring isolation for atrial fibrillation ablation: how to do it and avoid the esophagus. HeartRhythm Case Rep. 2020;6(4):169–73. 32322489 10.1016/j.hrcr.2019.09.010 PMC7156984

[13] Thiyagarajah A, Mahajan R, Iwai S, et al. Single ring isolation for atrial fibrillation ablation: impact of the learning curve. J Cardiovasc Electrophysiol. 2022;33(4):608–17. 35077605 10.1111/jce.15387

[14] Shin DG, Ahn J, Han SJ, Lim HE. Efficacy of high-power and short-duration ablation in patients with atrial fibrillation: a prospective randomized controlled trial. Europace. 2020;22(10):1495–501. 32810203 10.1093/europace/euaa144

[15] Wewers ME, Lowe NK. A critical review of visual analogue scales in the measurement of clinical phenomena. Res Nurs Health. 1990;13(4):227–36. 2197679 10.1002/nur.4770130405

[16] Kim TH, Park J, Uhm JS, Joung B, Lee MH, Pak HN. Pulmonary vein reconnection predicts good clinical outcome after second catheter ablation for atrial fibrillation. Europace. 2017;19(6):961–7. 27256420 10.1093/europace/euw128

[17] Lim TW, Koay CH, McCall R, See VA, Ross DL, Thomas SP. Atrial arrhythmias after single-ring isolation of the posterior left atrium and pulmonary veins for atrial fibrillation: mechanisms and management. Circ Arrhythm Electrophysiol. 2008;1(2):120–6. 19808402 10.1161/CIRCEP.108.769752

[18] Kurin M, Fass R. Management of gastroesophageal reflux disease in the elderly patient. Drugs Aging. 2019;36(12):1073–81. 31541359 10.1007/s40266-019-00708-2

[19] Durazzo M, Campion D, Fagoonee S, Pellicano R. Gastrointestinal tract disorders in the elderly. Minerva Med. 2017;108(6):575–91. 28974085 10.23736/S0026-4806.17.05417-9

[20] Thomas SP, Lim TW, McCall R, Seow SC, Ross DL. Electrical isolation of the posterior left atrial wall and pulmonary veins for atrial fibrillation: feasibility of and rationale for a single-ring approach. Heart Rhythm. 2007;4(6):722–30. 17556191 10.1016/j.hrthm.2007.01.034

[21] Thiyagarajah A, Mahajan R, Iwai S, et al. Single ring isolation with inferior line sparing for atrial fibrillation: a proof-of-concept study. Circ Arrhythm Electrophysiol. 2021;14(4):e009552. 33858182 10.1161/CIRCEP.120.009552

[22] Bourier F, Duchateau J, Vlachos K, et al. High-power short-duration versus standard radiofrequency ablation: insights on lesion metrics. J Cardiovasc Electrophysiol. 2018;29(11):1570–5. 30168230 10.1111/jce.13724

[23] Jin S, Fu L, Jiang J, et al. Comparison of effectiveness and safety between high-power short-duration ablation and conventional ablation for atrial fibrillation: a systematic review and meta-analysis. J Interv Cardiol. 2022;2022:6013474. 36072362 10.1155/2022/6013474 PMC9398879

[24] Han HC, Ha FJ, Sanders P, et al. Atrioesophageal fistula: clinical presentation, procedural characteristics, diagnostic investigations, and treatment outcomes. Circ Arrhythm Electrophysiol. 2017;10(11):e005579. 29109075 10.1161/CIRCEP.117.005579

[25] Weerasooriya R, Khairy P, Litalien J, et al. Catheter ablation for atrial fibrillation: are results maintained at 5 years of follow-up. J Am Coll Cardiol. 2011;57(2):160–6. 21211687 10.1016/j.jacc.2010.05.061

[26] Parkash R, Tang AS, Sapp JL, Wells G. Approach to the catheter ablation technique of paroxysmal and persistent atrial fibrillation: a meta-analysis of the randomized controlled trials. J Cardiovasc Electrophysiol. 2011;22(7):729–38. 21332861 10.1111/j.1540-8167.2011.02010.x

[27] Lim TW, Koay CH, See VA, et al. Single-ring posterior left atrial (box) isolation results in a different mode of recurrence compared with wide antral pulmonary vein isolation on long-term follow-up: longer atrial fibrillation-free survival time but similar survival time free of any atrial arrhythmia. Circ Arrhythm Electrophysiol. 2012;5(5):968–77. 22972873 10.1161/CIRCEP.111.970293

[28] Verma A, Jiang CY, Betts TR, et al. Approaches to catheter ablation for persistent atrial fibrillation. N Engl J Med. 2015;372(19):1812–22. 25946280 10.1056/NEJMoa1408288

